# Globally Normal Bistable Motion Perception of Anisometropic Amblyopes May Profit From an Unusual Coding Mechanism

**DOI:** 10.3389/fnins.2018.00391

**Published:** 2018-06-07

**Authors:** Jiachen Liu, Yifeng Zhou, Tzvetomir Tzvetanov

**Affiliations:** ^1^Hefei National Laboratory for Physical Sciences at Microscale, School of Life Science, University of Science and Technology of China, Hefei, China; ^2^State Key Laboratory of Brain and Cognitive Science, Institute of Biophysics, Chinese Academy of Science, Beijing, China; ^3^Anhui Province Key Laboratory of Affective Computing and Advanced Intelligent Machine and School of Computer and Information, Hefei University of Technology, Hefei, China

**Keywords:** plaid motion, anisometropic amblyopia, motion coding mechanism, bistable percept, model prediction

## Abstract

Anisometropic amblyopia is a neurodevelopmental disorder of the visual system. There is evidence that the neural deficits spread across visual areas, from the primary cortex up to higher brain areas, including motion coding structures such as MT. Here, we used bistable plaid motion to investigate changes in the underlying mechanisms of motion integration and segmentation and, thus, help us to unravel in more detail deficits in the amblyopic visual motion system. Our results showed that (1) amblyopes globally exhibited normal bistable perception in all viewing conditions compared to the control group and (2) decreased contrast led to a stronger increase in percept switches and decreased percept durations in the control group, while the amblyopic group exhibited no such changes. There were few differences in outcomes dependent upon the use of the weak eye, the strong eye, or both eyes for viewing the stimuli, but this was a general effect present across all subjects, not specific to the amblyopic group. To understand the role of noise and adaptation in such cases of bistable perception, we analyzed predictions from a model and found that contrast does indeed affect percept switches and durations as observed in the control group, in line with the hypothesis that lower stimulus contrast enhances internal noise effects. The combination of experimental and computational results presented here suggests a different motion coding mechanism in the amblyopic visual system, with relatively little effect of stimulus contrast on amblyopes' bistable motion perception.

## Introduction

Amblyopia is a neurodevelopmental disorder of the visual system. The condition is caused by an imbalance in visual input during cortex development, mostly in infancy (Wong, 2012; Hess and Thompson, 2015). Anisometropic amblyopia is typically due to the presence of a chronic blur. These conditions result in a weakening or suppression of the input from the amblyopic eye, and, thus, this input is processed abnormally within the visual cortex (Hubel and Wiesel, 1965, 1970; Kiorpes and McKee, 1999; Hess and Thompson, 2015). Such an abnormal processing causes amblyopes to see differently from neurotypical subjects in visual perception tasks; for example, amblyopes may exhibit a reduction in contrast sensitivity, stereo-acuity (3D, depth perception), or visual acuity (Bradley and Freeman, 1981; Levi et al., 2011). In contrast, suprathreshold contrast perception seems equivalent between both eyes of amblyopes (Hess and Bradley, 1980), while prolonged observations of static gratings by amblyopes make them report illusory static or dynamic patterns in the stimulus (Sireteanu et al., 2008; Thiel and Iftime, 2016).

In addition to the above basic visual features, other spatial and temporal processing are also affected by amblyopia in early visual cortices (Barnes et al., 2001; Bonhomme et al., 2006; Hess et al., 2010; Li et al., 2011). Increasing evidence has demonstrated that amblyopia is also associated with abnormal function of the MT/MST areas, which are highly motion-sensitive and related to local and global motion integration (Britten et al., 1992; Born and Bradley, 2005; Majaj et al., 2007). There is strong neurophysiological evidence to suggest that motion integration and segregation processing involve area MT (Newsome and Parés, 1988; Salzman et al., 1990). In addition, psychophysical studies have shown abnormal global motion perception in amblyopia, even after adjusting for the deficits in contrast sensitivity. These results strongly suggest that the motion-sensitive areas MT/MST are affected by this disorder (Ellemberg et al., 2002; Constantinescu et al., 2005; Simmers et al., 2006; Aaen-Stockdale et al., 2007; Thompson et al., 2008; Ho and Giaschi, 2009; El-Shamayleh et al., 2010), and a recent neuroimaging study found evidence of abnormal cortical processing of pattern motion in amblyopia (Thompson et al., 2012).

In psychophysical research, plaid motion is a particular stimulus used to investigate the underlying neural mechanisms of motion integration and segregation (Adelson and Movshon, 1982). Plaid stimuli are typically constructed from two drifting gratings within a circular aperture. The drifting directions of both gratings are different. When the two gratings have similar temporal and spatial properties, the stimulus will produce an initial percept of a single patterned surface drifting in a “global” direction, which is a unique combination of both component directions. With prolonged observation of the pattern, a perceptual switching phenomenon occurs; the plaid motion can be seen either as “coherent motion” (a single object moving rigidly) or as “transparent motion” (two independent gratings sliding over each other), dubbed bistable motion perception. Because of the advances in the theoretical understanding of bistable perception, we considered that plaid motion would be a particularly useful probe for investigating the mechanisms of motion segmentation and integration and help us to unravel in more detail the deficits in the amblyopic visual motion system.

The various observations of bistable perception have inspired models of multistability, which mainly focus on bistable rivalry (Lago-Fernández and Deco, 2002; Laing and Chow, 2002; Moreno-Bote et al., 2007). In such models, the random alternation of percepts is influenced by the competition between two neuronal populations via reciprocal inhibition, noise levels in the neural inputs and some sort of adaptation, e.g., spike frequency adaptation and/or synaptic depression. Such models are extendable to tristable percepts, of which plaid motion perception is argued to be an example (Huguet et al., 2014). In all of these models, the exact number of percept switches together with the durations of the two major types of percepts are very sensitive to internal variables, especially internal noise. Thus, any changes in internal variables differentially affect all measurable variables.

This manuscript first describes results of three experiments performed to compare the bistable motion perception in anisometropic amblyopes (AMB) and neurotypical observers (NTE). Experiment 1 was mainly performed as an exploratory study to search for plausible differences between AMB and NTE in plaid motion perception. This experiment led to the hypothesis of differential effects associated with stimulus strength between AMB and NTE that was tested in Experiment 2. Experiment 3 was a control test of the main finding of contrast effects. In the last part, with the help of simulations, we analyzed one model predictions (Moreno-Bote et al., 2007) in order to compare to the experimental results, and thus to propose putative changes in the mechanisms of motion coding in the amblyopic visual system.

## Methods

### Observers

A total of 32 observers participated in the experiments, including 17 normal-sighted subjects (five women and 12 men; including two authors; age range 20–42) and 15 anisometropic amblyopes (one woman and 14 men; age range: 23–27). A portion of the observers in these two groups participated in experiments 1, 2, and 3. The exact number of subjects within a given experiment is stated in the corresponding section. All amblyopes had anisometropic amblyopia; amblyope #10 had bilateral amblyopia. For that person, the eye with the best visual acuity (strong eye) was treated as the fellow eye in all the analysis. Detailed ophthalmologic characteristics of these observers, including amblyopia type and optical correction, were obtained during normal university medical examinations at the department of ophthalmology in the hospital of USTC. The amblyopic group was defined according to the Preferred Practice Protocol (PPP) of The American Academy of Ophthalmology (Wallace et al., 2018), with anisometropic type was defined as the difference of dioptre sphere above 1.5 and/or the difference of dioptre of cylinder over 1.0 who can not fuse image in retina well binocularly. Nonamblyopes had normal or corrected-to-normal eyesight, while amblyopes wore their best refractive corrections. All observers provided informed consent and received a fee of 60 CNY/hour for participating in the experiments. The experiments were approved by the ethics committee of the School of Life Science of USTC and followed the tenets of the Declaration of Helsinki for experiments with human subjects. Table 1 presents the eyes characteristics of the amblyopes.

**Table 1:** Ophthalmic details of the observers with amblyopia.

**Obs**	**Age/sex**	**Type**	**Refraction**	**SA**	**VA (MAR)**
Amb1	25/M	RE anis	+6.00 DS/+1.00 DCx25	100	10.00
		LE	Ø		1.000
Amb2	27/M	RE anis	+4.00 DS/+1.00 DCx85	50	10.00
		LE	Ø		1.00
Amb3	26/M	RE anis	+2.50 DS/+1.00 DCx170	160	3.16
		LE	−0.75 DS		0.63
Amb4	23/F	RE anis	−2.00 DCx110	100	2.00
		LE	−6.00 DS		1.00
Amb5	26/M	RE anis	+1.50 DS/+1.50 DCx60	400	6.31
		LE	−1.00 DS/−0.50 DCx160		1.00
Amb6	23/M	RE anis	+1.00 DCx105	400	3.98
		LE	−4.00 DS/−1.25 DCx30		0.79
Amb7	25/M	RE	−0.500 DS	25	1.00
		LE anis	+2.500 DS/0.500 DCx90		1.58
Amb8	25/M	RE	+3.00 DS/+1.00 DCx85	400	0.79
		LE anis	+5.50 DS/0.75 DCx95		3.16
Amb9	23/M	RE	−1.250 DS/−1.00 DCx160	63	0.79
		LE anis	1.00 DCx80		1.58
Amb10	23/M	RE anis	−5.50 DS/−2.00 DCx10	32	3.98
		LE anis	−5.250 DS/-5.00 DCx175		6.31
Amb11	25/M	RE	−1.50 DS/−0.50 DCx30	400	0.79
		LE anis	+4.50 DS/0.50 DCx35		5.01
Amb12	25/M	RE	−2.75 DS/−0.50 DCx10	400	1.00
		LE anis	+1.00 DS/+1.00 DCx95		3.98
Amb13	24/M	RE	−2.75 DS/−1.00 DCx20	50	1.00
		LE anis	+3.75 DS/0.75 DCx115		2.00
Amb14	26/M	RE anis	−2.50 DS	100	3.16
		LE	+2.00 DS/+0.50 DCx96		0.63
Amb15	26/M	RE anis	+1.00 DS/+1.50 DCx95	32	3.98
		LE	Ø		0.79

*Obs, observer; Amb, anisometropic amblyope; M, male; F, female; RE, right eye; LE, left eye; anis, anisometropic; DS, dioptre sphere; DC, dioptre of cylinder; Ø, plano; SA, stereo acuity; VA, visual acuity; MAR, minimum angle of resolution*.

### Apparatus

Stimuli were presented on an ASUS VG248 monitor with a 1,920 × 1,080-pixel resolution at a frame rate of 120 Hz. Observers were comfortably seated 100 cm in front of the screen in a dark room, with their chin and forehead resting on a chinrest. When the eye signal was available, binocular or monocular eye movements (randomly) were monitored and recorded for a portion of the observers (13 amblyopes/10 normal observers) with an Eyelink 1,000 eye recording setup and sampled at 500 Hz to confirm correct eye fixation at the stimulus location.

### Stimuli

The stimulus comprised two rectangular-wave gratings presented through a circular aperture 7.7° in diameter on a middle-gray background of RGB 126. Gratings moved at 3°/s (defined in the direction normal to their orientation) in directions 90° apart (angle α hereafter), with a spatial frequency of 3 c/d and duty cycle of 50%. The mean direction of motion of both gratings was either vertical upward or horizontal leftward, thus making the coherent pattern perceived as moving upwards or leftwards, respectively. Grating contrast was defined in RGB units, and two contrasts of 30% (high) and 5% (low) values were possible, with both gratings having the same contrast. A pink fixation point was added in the middle of the circular aperture to help subjects locate the stimulus center and minimize optokinetic nystagmus (Huguet et al., 2014), and subjects were instructed to fixate this point throughout the stimulus presentation.

### Experimental procedure

Subjects were first familiarized with the stimuli and procedure. They had to report the time of percept change with two keyboard keys, with each key indicating that they perceived either coherent motion or transparent motion. They were instructed to passively report the percepts, without trying to influence them. Each observer was exposed to both global coherent directions (upward and leftward) to avoid motion direction adaptation, one (Experiment 1) or two (Experiment 2) contrast levels (for Experiment 1, 30% contrast; for Experiment 2, 30 and 5% contrast), and three eye conditions (binocular, left, right eye monocular), corresponding to a total of 6 or 12 different stimulus configurations. Presentation time was 120 s for each stimulus, and observers were tested on each configuration one time. The order of presentation was random. Because the first percept is known to always be coherent in normal-sighted observers (Hupé and Rubin, 2003), and amblyopes are able to demonstrate possible grating misperceptions/illusions (Hess et al., 1978; Hess and Bradley, 1980; Thompson et al., 2008; Thiel and Iftime, 2016), each observer was debriefed at the end of each 120-s trial about their first percept (coherent or not) and overall visibility of the pattern. All participants reported that they could clearly see the stimuli, a single moving plaid stimulus and two grating surfaces sliding over each other, in all conditions, even at the lowest contrast used in this study. No amblyopes reported differences between AE and fellow eye perception of the moving gratings, out of the switch rate/duration differences. The dominant eye of each subject was assessed with the hole-in-card experiment. Stereo acuity was assessed with the Titmus Stereopsis Test. Visual acuity was measured using a standard wall-mounted Tumbling E chart, from a distance of 5 metres, and defined as the score associated with a correct judgment rate of 75% at the minimum angle of resolution.

### Model simulation and numerical procedures

We implemented the tristable model of motion coherence/transparency proposed by Huguet et al. (2014). This model is a firing rate-based tristable model that includes three pools of neuronal populations that encode three different percepts: coherence (C), transparent with the leftward moving grating on top (*T*_*L*_), and transparent with the rightward moving grating on top (*T*_*R*_). The equations describing the dynamics of the three populations are:

(1) $$\begin{array}{l} \tau \frac{dr_{c}}{dt}=\;-r_{c}+S\left(-\beta_{1}r_{T_{R}}-\;\beta_{1}r_{T_{L}}-\;a_{c}+\;I_{c}+\;n_{c}\right)\\ \tau \frac{dr_{T_{R}}}{dt}=\;-r_{T_{R}}+S\left(-\beta_{1}r_{c}-\;\beta_{2}r_{T_{L}}-\;a_{T_{R}}+\;I_{T_{R}}+\;n_{T_{R}}\right)\\ \tau \frac{dr_{T_{L}}}{dt}=\;-r_{T_{L}}+S\left(-\beta_{1}r_{c}-\;\beta_{2}r_{T_{R}}-\;a_{T_{L}}+\;I_{T_{L}}+\;n_{T_{L}}\right) \end{array}$$

with *a*_*i*_, *I*_*i*_, and *n*_*i*_ representing adaptation, external input, and noise for each population, respectively. The time constant τ was τ = 10 ms. β_1_ is the cross-inhibition strength between population C and T (including *T*_*R*_ and *T*_*L*_), while β_2_ is the inhibition strength between *T*_*R*_ and *T*_*L*_. The intensity of external input changes is represented with *I*_*C*_ and *I*_*T*_ = *I*_*T*_*R*__ = *I*_*T*_*L*__.

The function S is a sigmoidal transducer of input-output function:

(2) $$\begin{array}{l} S\left(x\right)=\frac{1}{1+\theta^{-\left(x-\theta \right)/k}} \end{array}$$

with threshold θ = 0.2 and *k* = 0.1.

The adaptation of firing activity was done through the terms *a*_*C*_, *a*_*T*_*R*__, *a*_*T*_*L*__ and all followed the same time evolution:

(3) $$\begin{array}{l} \tau \frac{da_{i}}{dt}=-a_{i}+\gamma r_{i} \end{array}$$

with τ = 2,500 ms, and a maximum strength of γ = 0.25 for all populations.

Noise input is modeled with an Ornstein-Uhlenbeck process as:

(4) $$\begin{array}{l} \frac{dn_{i}}{dt}=-\frac{n_{i}}{\tau_{s}}+\sigma \sqrt{\frac{2}{\tau_{s}}}\times \xi \left(t\right) \end{array}$$

with τ_*S*_ = 200 ms, σ = 0.08, and ξ(*t*) is a white-noise process whose mean value is zero with a standard deviation of one and no temporal correlations.

In this model (Huguet et al., 2014), we adjusted the cross-inhibition strength values β_1_ and β_2_, external input value *I*_*C*_ and *I*_*T*_ (*I*_*T*_*R*__ and *I*_*T*_*L*__ were set equal), noise strength value σ, and adaptation strength value γ to reproduce our behavioral results with other parameters remaining unchanged. The time window of simulations was set to 120 s, corresponding to the length of one block of measure in the psychophysical experiment, and repeated simulations were performed to obtain the mean and variability of the variables analyzed in the experiments.

Since we focused on the bistable condition, we report only transparent and coherent states by considering *T*_*R*_ and *T*_*L*_ as the transparent percept. A coherent percept was defined when *r*_*C*_ was simultaneously higher than *r*_*T*_*R*__ and *r*_*T*_*L*__ and otherwise defined as transparent. For each 120 s of simulations, we computed the number of switches and durations of coherent and transparent states.

### Data analysis

For each 120-s trial, the number of percept changes was computed from the first report of a transparent percept to the end of the trial, as in work by Hupé and Rubin (2003). The dominance durations were measured between successive presses of the two keys. The duration of the last interrupted percept was not computed. The first percept was coherent in all trials (as reported in the debriefing), but in some conditions, a few subjects did not first press the “coherent” percept key, due to their knowledge of this appearance. Dominance durations were log10-transformed (Moreno-Bote et al., 2010).

Each dependent variable was analyzed with within-between analysis of variance, while all statistical levels used Geisser-Greenhouse epsilon-hat-adjusted values where appropriate. In the first analysis, the dependent variable was the number of key-presses for each condition, which allowed for the comparison of the frequencies of perception switches in different conditions and observers (amblyopes/normal observers). This analysis included the data from all subjects. In the second analysis, the dependent variable was the mean duration of the percept, with an additional within-subject factor in the ANOVA corresponding to coherent and transparent conditions. In this analysis, observers who were unable to see perceptual switches in at least one condition were not included due to lack of the corresponding variable. This phenomenon only appeared in 3 out of 15 anisometropic amblyopes (2 in Experiment 1 and 2 in Experiment 2) and 1 out of 17 NTE subjects (in Experiment 1), and it was mostly present for horizontal motion directions. We also calculated the mean value and standard deviation for each condition across all normal subjects and found that 1 of the 11 subjects in Experiment 2 had percept durations that deviated above 2 *SD* from the between-subjects mean of the condition in 8 out of 24 conditions. In contrast, the other subjects had such deviations in a maximum of 2 conditions. For this reason, we also removed this subject data in the analysis of percept durations.

## Results

### Experiment 1

In the first experimental test, we measured the performance of each subject in three eye conditions (binocular, monocular with strong eye, and monocular with weak eye) with only a strong contrast of the gratings (30%) and global moving directions upwards and leftwards. We focused on the number of perceptual switches and mean duration of each percept type. Twenty subjects participated in this experiment; 10 of them were anisometropic amblyopes (AMB), and the remaining were neurotypical subjects (including two authors) that had no known visual deficits (NTE). During the experiment, all amblyopic subjects reported that they did not feel any difference between the fellow eye or binocular condition when using the amblyopic eye to watch the stimulus.

#### Frequency of perceptual switches

**Figure 1:**
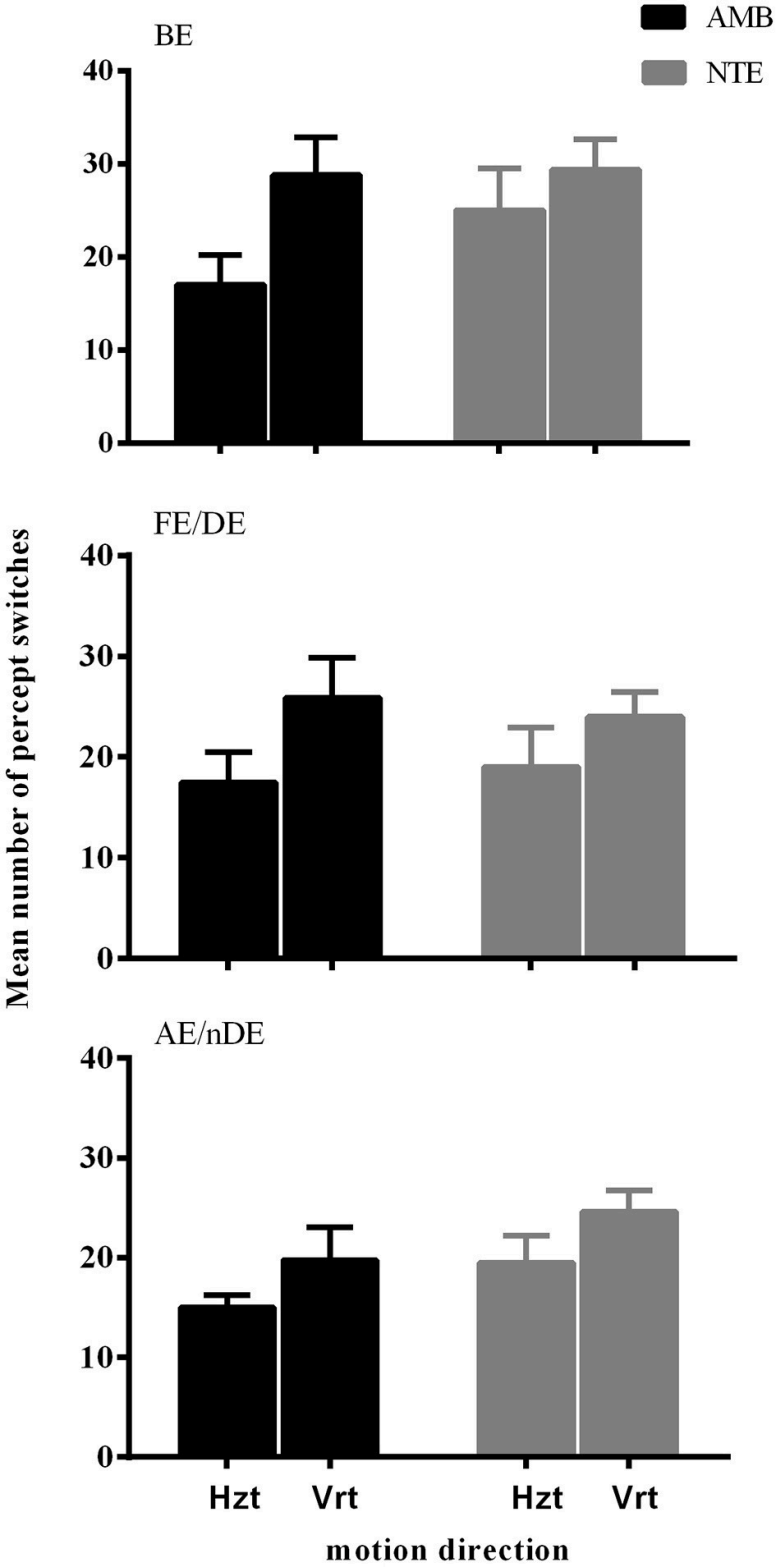
The mean number of percept switches in Experiment 1, split between the factors motion direction (Hzt: horizontal, Vrt: vertical), eye viewing (BE-binocular, AE/nDE-nondominant eye, FE/DE- fellow/dominant eye), and Group (AMB/NTE). Error bars indicate between-subject SEM.

Figure 1 illustrates the number of key-presses in each viewing condition for the two groups. There was a significant difference between the two moving directions [*F*_(1, 18)_ = 15.865, *p* = 0.001] showing that, globally, the number of perceptual switches for the vertical motion directions were higher than for the horizontal directions. Eye viewing conditions also showed significant differences in perceptual switches [*F*_(1.987, 35.758)_ = 5.836, *p* = 0.006], with the *post-hoc* Bonferroni test revealing a difference between the binocular and weak eye conditions [*F*_(1, 18)_ = 10.860, *p* = 0.004]. Statistical analysis showed that there was no difference between the two groups of subjects [*F*_(1, 18)_ = 1.061, *p* = 0.317], nor a significant interaction between the observer groups and the other factors (see Table 2 for full ANOVA results).

**Table 2:** ANOVA results on Presses Number of Experiment 1.

**Variables**	**df**	***F***	**Sig**.	**Partial Eta Squared**
Eye	1.987, 35.758	5.836	0.006	0.245
Eye * Group	1.987, 35.758	1.988	0.152	0.099
Dir	1, 18	15.865	0.001	0.468
Dir * Group	1, 18	1.146	0.298	0.060
Eye * Dir	1.736, 31.241	3.141	0.064	0.149
Eye * Dir * Group	1.736, 31.241	1.319	0.279	0.068
Group	1, 18	1.601	0.317	0.056

#### Duration of the two percept types in different conditions

**Figure 2:**
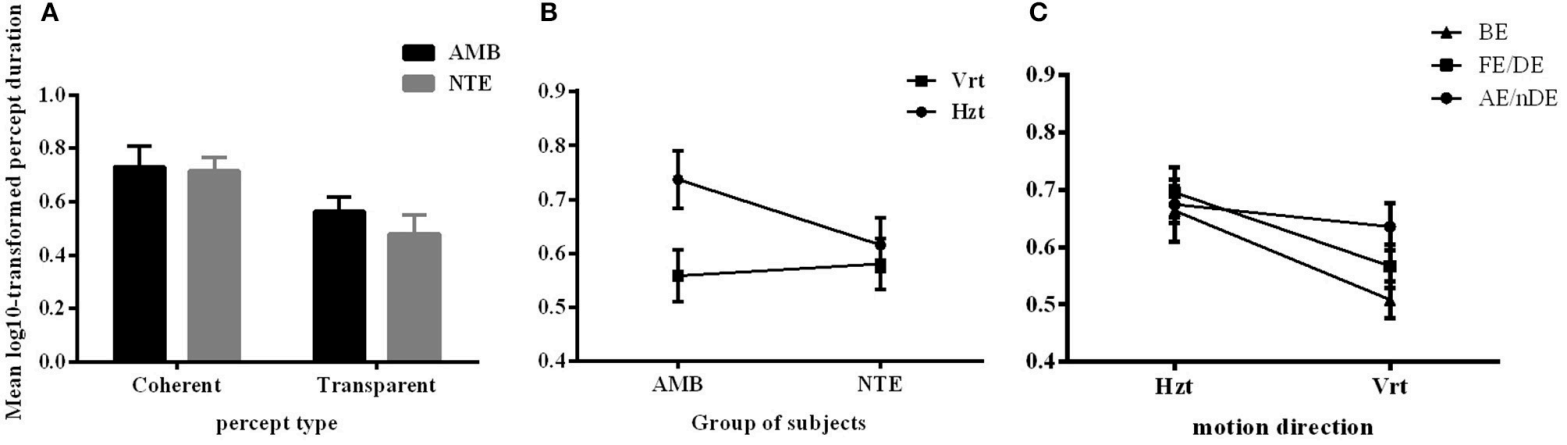
The mean log10-transformed percept durations showing **(A)** a main effect of percept type, **(B)** main effect of motion direction, and **(C)** an interaction between eye condition and direction. Mean of the log10-transformed percept durations expressed in seconds. C, coherent; T, transparent; NTE, Neurotypical/Normal; AMB, Amblyopes; Vrt, vertical; Hzt, horizontal; BE, binocular condition; AE/nDE, weak eye of subjects; FE/DE, fellow/dominant eye of subjects. Error bars indicate between-subject SEM.

Figure 2 summarizes the results of the duration of the percepts. Statistical analysis showed that there was no difference between the two groups of subjects [*F*_(1, 15)_ = 0.559, *p* = 0.466], indicating that the mean perceived duration of each percept type was similar in normal and amblyopic people. A significant difference was found in the durations of each percept type [*F*_(1, 15)_ = 10.925, *p* = 0.005], with duration of coherent percept being longer than the duration of the transparent percept, independent of the subject group (see Figure 2A). We also found significant differences in motion direction [*F*_(1, 15)_ = 22.272, *p* < 0.001] with the mean of log10-transformed duration of horizontal direction being longer than that of the vertical direction (mean of horizontal = 0.673, mean of vertical = 0.570) and a significant interaction between direction and group [*F*_(1, 15)_ = 10.062, *p* = 0.006; see Figure 2B]. This last interaction was due to the much longer percept duration for the horizontal motion directions than for the vertical ones in AMB, while NTE exhibited similar values for both directions. There was also an interaction between eye condition and direction [*F*_(1.927, 28.906)_ = 3.927, *p* = 0.031; Figure 2C]. For the horizontal direction, the means of the log10-transformed durations for each eye condition were similar but were distinct when the global motion direction was vertical. This difference may indicate that there are different strategies to address different motion directions. Additionally, with the change in the direction, the weak eye showed a relatively stable log10-transformed duration. *Post-hoc* Bonferroni-adjusted comparisons showed a difference between the weak eye and binocular condition in its interaction with direction [*F*_(1, 15)_ = 8.787, *p* = 0.01]. No significant differences were found in other factors (see Table 3 for complete ANOVA results).

**Table 3:** ANOVA results on Mean of log-10 Durations of Experiment 1.

**Variables**	**df**	***F***	**Sig**.	**Partial Eta Squared**
Per	1, 15	10.925	0.005	0.412
Per * Group	1, 15	0.297	0.594	0.019
Eye	1.869, 28.038	2.723	0.086	0.154
Eye * Group	1.869, 28.038	0.836	0.437	0.053
Dir	1, 15	22.272	0.000	0.598
Dir * Group	1, 15	10.062	0.006	0.401
Per * Eye	1.772, 26.586	0.722	0.479	0.046
Per * Eye * Group	1.772, 26.586	0.371	0.669	0.024
Per * Dir	1, 15	1.164	0.298	0.072
Per * Dir * Group	1, 15	0.033	0.858	0.002
Eye * Dir	1.927, 28.906	3.927	0.032	0.207
Eye * Dir * Group	1.927, 28.906	2.100	0.142	0.123
Per * Eye * Dir	1.740, 26.101	1.175	0.319	0.073
Per * Eye * Dir * Group	1.740, 26.101	0.468	0.605	0.030
Group	1, 15	0.559	0.466	0.036

### Experiment 2

From the above Experiment 1 results, we observed that there were few differences between amblyopes and non-amblyopes in their perception of a bistable plaid motion stimulus. This outcome was unexpected because, based on previous reports of stronger noise in the motion amblyopic system (Simmers et al., 2006) and possibly a very different visual motion coding system in amblyopes (Thompson et al., 2012), we expected that motion rivalry, due to its keen sensitivity to internal noise and inhibition strength (Huguet et al., 2014), would result in strong systematic differences between the two observer types. Given the non-significant differences, we realized that our experimental design might have missed the effects because of the relatively high contrast of the gratings. Thus, if the activation of the motion system was too high such that the signal-to-noise (SNR) ratio was relatively large, then any internal noise differences might have gone unnoticed. Therefore, we performed a second experiment that was identical to the first in all aspects except that one more factor was added, the contrast of the stimuli, with two levels, high (30%) and low (5%) contrast. By decreasing the contrast, we expected that the SNR would also decrease, and differences between the groups would be observed, with a prediction that there would be a main effect of lower contrast in which the low-contrast condition would be associated with more perceptual switches in amblyopes when compared to the high-contrast condition.

Twenty-one subjects participated in this experiment, with 10 anisometropic amblyopes (AMB; 5 of them also participated in Experiment 1), and the remaining were neurotypical subjects (NTE; 4 of them participated in Experiment 1).

#### Frequency of perceptual switches

**Figure 3:**
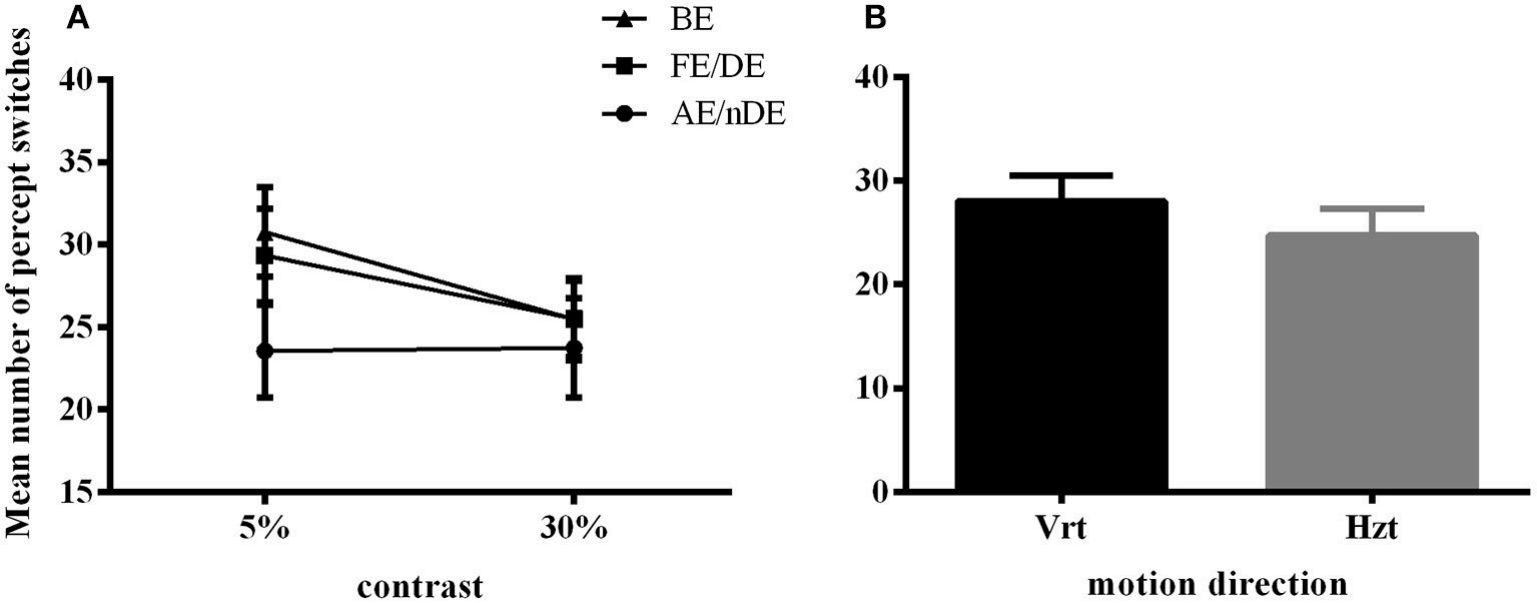
Main significant effects and interaction in Experiment 2 for number of percept switches. **(A)** Interaction plot for contrast and eye conditions. DE (dominant eye) and FE (fellow eye) corresponded to the strong eye for normal and amblyope observers, respectively; nDE (non-dominant eye) and AE (amblyopic eye) corresponded to the weak eye for normal and amblyope observers, respectively. BE was the binocular condition. **(B)** Main effect of global motion direction. Mean number of switches was higher in the vertical condition (Vrt) than in the horizontal condition (Hzt). Error bars indicate between-subjects SEM.

Here, we still used the number of key-presses to represent the frequency of perceptual switches. Analysis included data from all 21 subjects (10 AMB and 11 NTE). Figure 3 shows the main significant effects and interaction of how the press number increased with lower contrast and that the frequency of percept switches was globally lower in the weak eye condition than in the other conditions. There was no significant difference in the performance of normal and amblyopic subjects [*F*_(1, 19)_ = 0.287, *p* = 0.598]. However, there was a significant difference in contrast [*F*_(1, 19)_ = 5.575, *p* = 0.029], direction [*F*_(1, 19)_ = 5.697, *p* = 0.028], and eye condition [*F*_(1.904, 36.171)_ = 4.446, *p* = 0.020]. The number of presses increased with the decrease in contrast, potentially due to an increase in internal noise or, equivalently, a decrease in the signal-to-noise ratio. Upon examination of the effects of the global direction of motion, both groups had higher percept switches when stimuli were moving upward (as in Experiment 1). *Post-hoc* comparisons (Bonferroni-corrected) for eye conditions showed a difference between the binocular and weak eye conditions [*F*_(1, 19)_ = 6.426, *p* = 0.02] and a difference between the weak and strong eye conditions [*F*_(1, 19)_ = 5.472, *p* = 0.03].

An interaction between contrast and eye condition was also found in this case [*F*_(1.904, 30.537)_ = 5.492, *p* = 0.013]. However, no other interactions were significant (see Table 4 for complete ANOVA results).

**Table 4:** ANOVA results on Presses Number of Experiment 2.

**Variables**	**df**	***F***	**Sig**.	**Partial Eta Squared**
Crt	1, 19	5.575	0.029	0.227
Crt * Group	1, 19	2.725	0.115	0.125
Dir	1, 19	5.697	0.028	0.231
Dir * Group	1, 19	2.954	0.102	0.135
Eye	1.904, 36.171	4.446	0.020	0.190
Eye * Group	1.904, 36.171	1.591	0.218	0.077
Crt * Dir	1, 19	1.911	0.183	0.091
Crt * Dir * Group	1, 19	0.019	0.893	0.001
Crt * Eye	1.607, 30.537	5.492	0.013	0.224
Crt * Eye * Group	1.607, 30.537	1.042	0.351	0.052
Dir * Eye	1.867, 35.469	1.550	0.227	0.075
Dir * Eye * Group	1.867, 35.469	0.046	0.946	0.002
Crt * Dir * Eye	1.828, 34.728	1.737	0.193	0.084
Crt * Dir * Eye * Group	1.828, 34.728	1.441	0.250	0.070
Group	1, 19	0.287	0.598	0.015

#### Duration of two percept types in different conditions

Here, we analyzed the duration of both percept types (i.e., coherent and transparent) for different contrast, eye, and moving direction conditions and whether there were differences between neurotypical subjects and anisometropic amblyopes; 2/10 AMB were not included because of at least one condition with no percept switch, and 1/11 NTE was excluded as an outlier (see section Methods).

**Figure 4:**
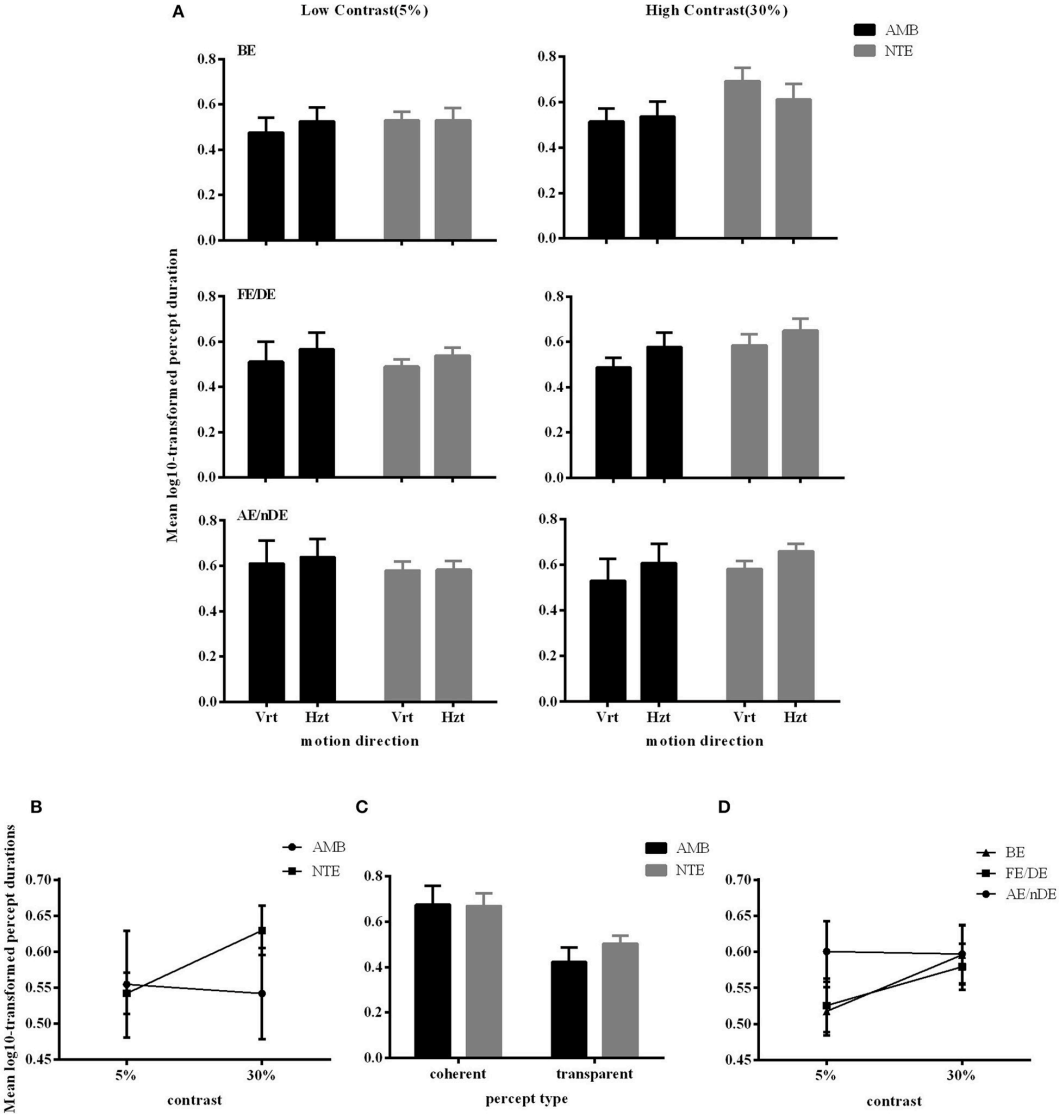
Main significant effects and interactions in Experiment 2 for variable percept durations. **(A)** Results of mean of log10-transformed percept durations expressed in seconds for eye condition, motion direction, and subject group. **(B)** Interaction between contrast and subject group. **(C)** Interaction between contrast and eye conditions. **(D)** Main effect of percept type. Note there was no difference between amblyopic and normal subjects. Error bars indicate between-subjects SEM.

Figure 4A illustrates the durations of both direction and eye conditions for subject groups and stimulus contrast conditions. Statistical analysis showed that there were no differences between the two groups of subjects [*F*_(1, 16)_ = 0.298, *p* = 0.593], indicating that globally, percept durations were similar in normal and amblyopic people. Significant differences were found across contrast conditions [*F*_(1, 16)_ = 5.173, *p* = 0.037] and percept type [*F*_(1, 16)_ = 19.241, *p* = 0.0005; Figures 4B,C]. Lower contrasts globally decreased percept duration, paralleling the increase in number of switches. The duration in the coherent percept was always longer than that in the transparent percept regardless of subject group (Figure 4C).

ANOVA also showed significant interactions between subject groups and contrast condition [group vs. contrast, *F*_(1, 16)_ = 9.326, *p* = 0.008; Figure 4B]. In NTE, percept duration decreased with a decrease in contrast, while amblyopes had no clear variation. This effect suggested that amblyopes seem to have a different motion processing mechanism from NTE. Another interaction showed a significant effect of the contrast and eye condition [*F*_(1.973, 31.575)_ = 4.420, *p* = 0.021; Figure 4D]. The performance in the binocular condition and stronger eye condition was similar across contrast conditions, while results differed according to contrast when the observer was using the weak eye to do the task. In this latter viewing condition, duration was slightly decreased when contrast increased, and the duration was always longer than the duration in the other two eye conditions. Thus, this interaction was mainly caused by the weak eye. *Post-hoc* Bonferroni-corrected comparisons for interaction between contrast and eye conditions showed that the dominant/fellow eye had a strong tendency for resulting in a different outcome than the binocular viewing condition [*F*_(1, 16)_ = 4.463, *p* = 0.051], while the weak eye had a different outcome than the binocular condition [*F*_(1, 16)_ = 7.624, *p* = 0.014]. No other effects were significant (Table 5).

**Table 5:** ANOVA results on Mean of log-10 Durations of Experiment 2.

**Variables**	**df**	***F***	**Sig**.	**Partial Eta Squared**
Crt	1, 16	5.173	0.037	0.244
Crt * Group	1, 16	9.326	0.008	0.368
Dir	1, 16	2.785	0.115	0.148
Dir * Group	1, 16	0.623	0.442	0.037
Eye	1.742, 27.868	3.155	0.064	0.165
Eye * Group	1.742, 27.868	1.524	0.236	0.087
Per	1, 16	19.241	0.000	0.546
Per * Group	1, 16	0.813	0.381	0.048
Crt * Dir	1,16	0.147	0.706	0.009
Crt * Dir * Group	1,16	0.074	0.789	0.005
Crt * Eye	1.973, 31.575	4.420	0.021	0.216
Crt * Eye * Group	1.973, 31.575	0.039	0.961	0.002
Dir * Eye	1.711, 27.375	2.178	0.139	0.120
Dir * Eye * Group	1.711, 27.375	0.545	0.559	0.033
Crt * Dir * Eye	1.783, 28.527	2.714	0.089	0.145
Crt * Dir * Eye * Group	1.783, 28.527	0.288	0.727	0.018
Crt * Per	1, 16	1.430	0.249	0.082
Crt * Per * Group	1, 16	0.682	0.421	0.041
Dir * Per	1, 16	0.360	0.557	0.022
Dir * Per * Group	1, 16	0.332	0.572	0.020
Crt * Dir * Per	1, 16	0.119	0.735	0.007
Crt * Dir * Per * Group	1, 16	0.400	0.536	0.024
Eye * Per	1.872, 29.944	2.213	0.130	0.121
Eye * Per * Group	1.872, 29.944	0.026	0.968	0.002
Crt * Eye * Per	1.799, 28.777	0.482	0.603	0.029
Crt * Eye * Per * Group	1.872, 29.944	1.325	0.279	0.076
Dir * Eye * Per	1.885, 30.154	0.256	0.763	0.016
Dir * Eye * Per * Group	1.885, 30.154	0.009	0.988	0.001
Crt * Dir * Eye * Per	1.607, 25.713	1.012	0.362	0.060
Crt * Dir * Eye * Per * Group	1.607, 25.713	0.925	0.390	0.055
Group	1, 16	0.298	0.593	0.018

### Experiment 3: control of contrast effects

**Figure 5:**
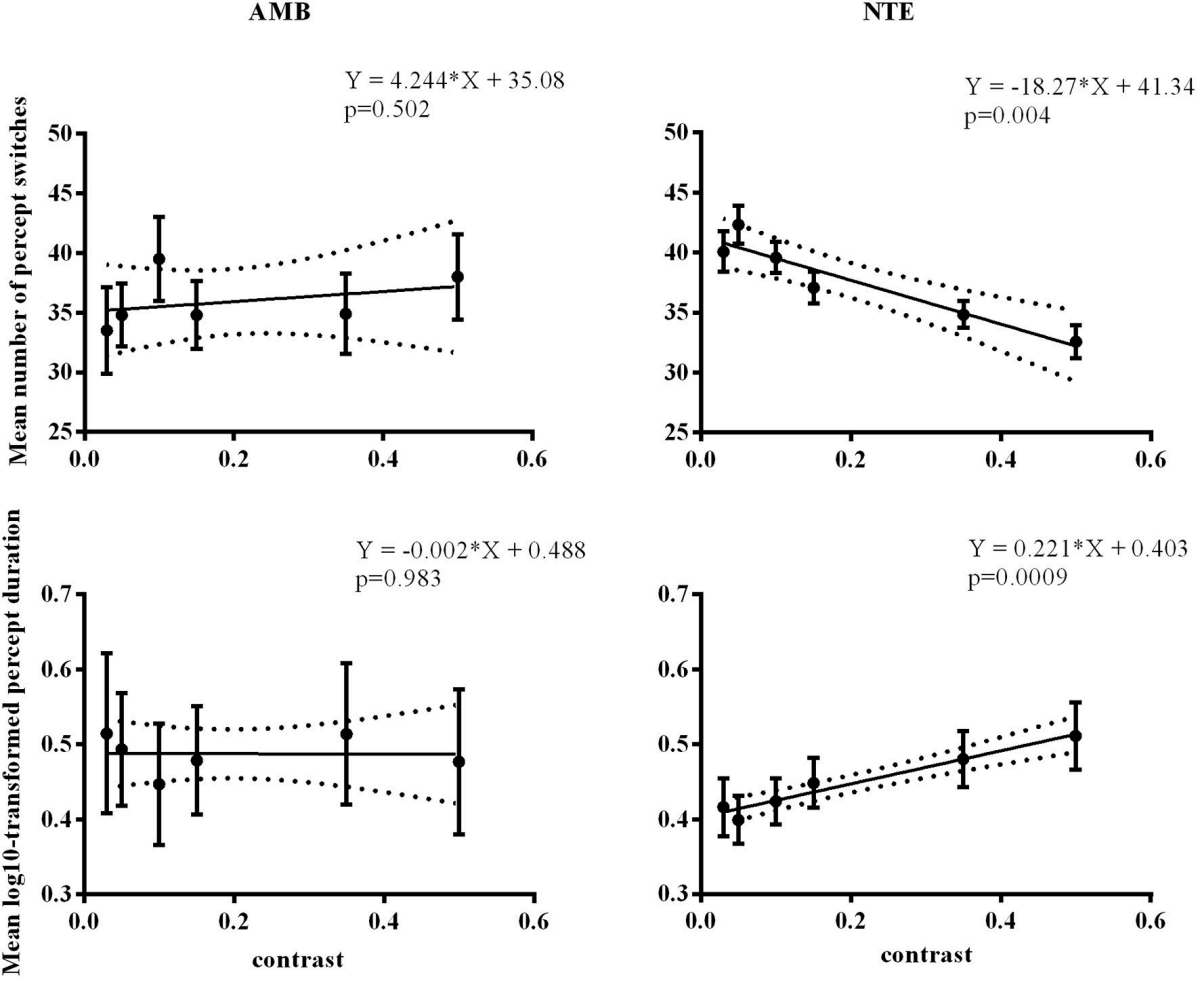
Linear regression across different contrast conditions in AMB and NTE. Top showing percept switches; bottom showing percept duration. Left column graphics for AMB; right column for NTE. Solid line was the best-fit line, while the dashed line indicates the 95% confidence band of the best-fit line. Error bar indicates the SEM.

We performed a control experiment to cross-check the effect of contrast in a different manner. We measured 5 AMB and 6 NTE (all participated in Experiment 1 or Experiment 2) in only the vertical condition to avoid a low number of switches with 6 levels of contrast (0.03, 0.05, 0.1, 0.15, 0.35, 0.5) with the hypothesis that the AMB should exhibit no variation with contrast, while the NTE should show an increase in the number of switches with a lower contrast. The results showed a clear interaction between the linear slopes of the number of switches versus contrast in AMB and NTE [group vs. contrast: *F*_(1, 8)_ = 11.9, *p* = 0.009], with the slope from AMB not different from zero (b = 4.2, CI = [−11.74, 20.22], *R*^2^ = 0.12, *p* = 0.502) and a significantly negative slope from NTE (*b* = −18.27, CI = [−26.76, 9.78], *R*^2^ = 0.90, *p* = 0.0039; see Figure 5). These results were also present when analyzing overall mean percept duration vs. contrast [Group vs. Contrast: *F*_(1, 8)_ = 9.31, *p* = 0.016; Figure 5]. The results were nearly identical when regressing in log-contrast space (number of switches vs. log-contrast, interaction group vs. contrast: *F*_(1, 8)_ = 11.898, *p* = 0.009; percept duration vs. log-contrast, interaction group vs. contrast: *F*_(1, 8)_ = 9.037, *p* = 0.017].

In summary, as expected, we found that contrast affected percept switches and percept durations by increasing the number of switches and decreasing the durations of the percepts with lower contrasts of gratings. In line with our expectation, this effect was mainly observed in NTE, and AMB showed no clear changes in percept duration with changes in contrast. Thus, based on our original hypothesis of decreased SNR with lower stimulus contrast, AMB seemed to show weak changes in plaid motion perception when contrast of the stimulus varied.

#### Correlation between bistability and VA or SA

We tested the correlation of the classic visual deficits as measured with the visual acuity (VA) and stereo acuity (SA) tests with the strength of bistability as measured through the number of switches. Table 6 shows that there were no significant correlations for all monocular conditions in the amblyopic group in both Experiments 1 and 2.

**Table 6:** Correlation between bistablity and SA or VA.

	**Number of percept switches vs**.	**Pearson Correlation**	**Sig**.	***N***
Experiment 1	Log of SA	−0.272	0.447	10
	Log of VA of AE	−0.192	0.596	10
	Log of VA of FE	0.217	0.546	10
Experiment 2	Log of SA	−0.034	0.927	10
	Log of VA of AE	−0.383	0.274	10
	Log of VA of FE	−0.372	0.290	10

#### Model predictions of bistable motion perception and consequences for the amblyopic visual motion system

**Figure 6:**
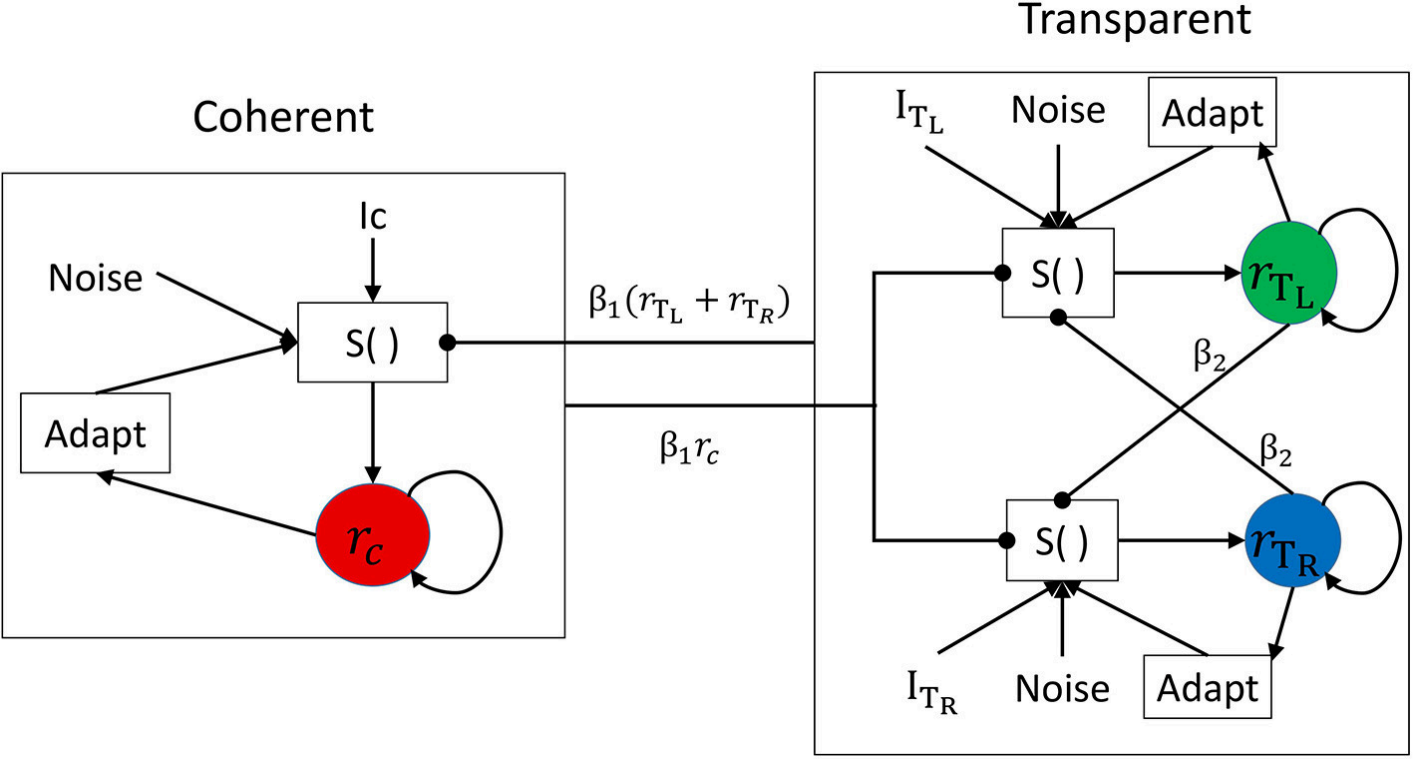
Network architecture for the neuronal competition model with direct mutual inhibition. The activity of each population is associated with a different percept: coherent (C), transparent right (T_R_), or transparent left (T_L_). Each population receives an excitatory deterministic input of strength I and independent noise *n*. Spike-frequency adaptation is present in each population. The function S() represents the sigmoidal transducer.

We used the tristable model defined by Huguet et al. (2014) to identify the plausible internal mechanisms underlying the results of Experiment 2. Because these authors argued and presented evidence that moving plaid stimuli consist of not two but three different percepts, i.e., the transparent condition with two clearly perceived sliding gratings can have two states with different depth orderings, and that there are perceptual switches across the three states, we considered this model as more relevant to our experiments even though the experimental task was only a simple dual report of either transparent or coherent motion. Their model incorporates three populations of neurons that code three possible percepts: coherence (C), transparent with the leftward (counterclockwise) moving grating on top (T_L_), and transparent with the rightward (clockwise) moving grating on top (T_R_); in the use of the model here, we considered the transparent state (T) only when the C state was not active. A schematic of the model is presented in Figure 6, and it contains 6 parameters (β_1_, β_2_, γ, σ, I_C_, I_T_ = I_TL_ = I_TR_). The model is used in a range of parameters providing winner-takes-all behavior where only one of the three populations can be active at a given time, thus representing the active percept. Competitive inhibition between the three neuronal populations, together with spike-frequency adaptation and internal noise, provide the substrate for perceptual switches between the percepts.

**Figure 7:**
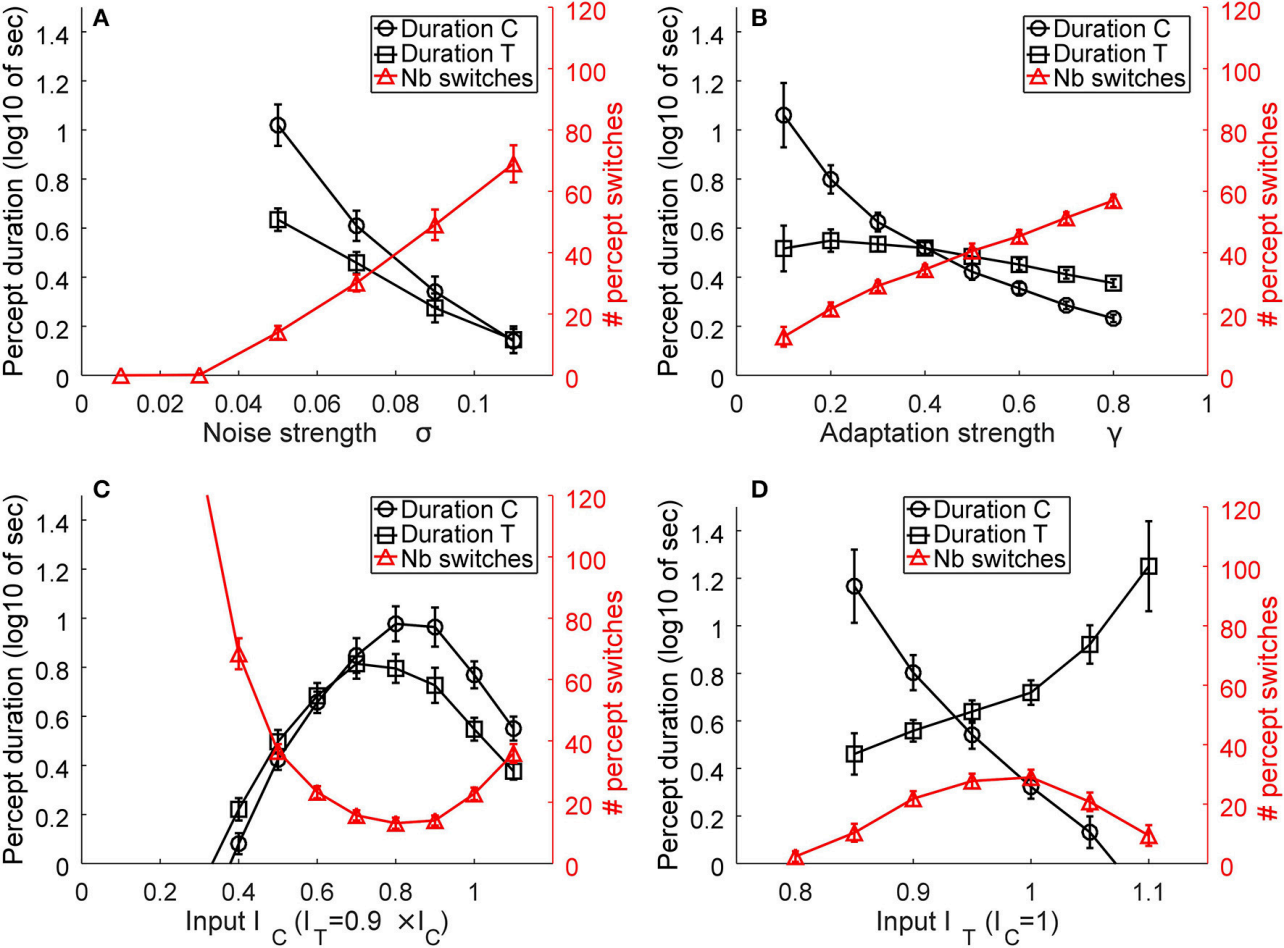
Representative model results. The effects of noise **(A)**, adaptation **(B)**, input strength, as the absolute value of I_C_ and I_T_ = I_TL_ = I_TR_ **(C)**, and relative transparent-to-coherent input **(D)** on the mean number of percept switches (red curves and right y-axis) and percept durations (black curves and left y-axis, expressed in log10 of seconds). Error bars indicate standard deviation of *n* = 30 simulations for each datum.

As described in Huguet et al. (2014), the model parameters play essential roles in determining the mean number of percept switches and their duration. We parametrically varied the parameters in order to understand their effects on the two main measures. Figure 6 presents representative simulation results for model parameters of β_1_ = 0.9, β_2_ = 0.7, σ = 0.06, γ = 0.2, I_C_ = 1, and I_T_ = I_TL_ = I_TR_ = 0.9I_C_, when varying one of the last four parameters. An increase in internal noise σ strongly increases the number of percept switches and concurrently decreases the durations of the two percepts of C and T states (Figure 7A). An increase in the adaptation strength γ also increases the number of perceptual switches but differentially affects the C and T states (Figure 7B), with the C state duration showing a stronger relation (decrease) to an increase in adaptation than the T state, making C durations longer than the T duration at low γ and the reverse pattern observed with stronger γ. When the input strength is varied (with relative input T-to-C as constant; Figure 7C), the number of percept switches rapidly decreases at low inputs, corresponding to rapid increases in the signal-to-noise ratio. However, the number of percept switches is also observed to exhibit a minimum after which it begins to increase again. From multiple simulations, we found that this minimum was strongly dependent on the relative input strengths (I_T_/I_C_) as well as on the inhibitory strengths (β_1_, β_2_; results not shown). The durations of the two types of percepts, C and T, concurrently changed with a strong change in the number of switches. The percepts also showed a change in their relative durations with low input strengths showing T states longer than C ones and a reversal at higher input values. Finally, a change in the relative strength between C and T inputs demonstrated a typical bell-shaped curve for the number of switches (Brascamp et al., 2015), with the maximum value near input equality, together with their concurrent C and T state duration changes (Figure 7D). These last effects mimicked the expected effects of relative input strengths onto the two variables as observed in previous reports (Moreno-Bote et al., 2010; Brascamp et al., 2015).

Similar observations were obtained for other inhibitory strengths (β_1_, β_2_) but with the absolute values of noise, input, adaptation, and relative input strengths correspondingly changed.

The above simulations show two important effects. First, the number of perceptual switches and percept durations are very sensitive to the internal noise and adaptation strength (Figures 7A,B). This observation supports the original hypothesis that plaid gratings would show differences between the two groups of subjects that putatively have different noise levels in their motion visual system (Mansouri and Hess, 2006). In contrast to this prediction, Experiment 1 did not show any differences between AMB and NTE. Second, a striking effect was present in the simulation for the absolute input strengths I_C_ and I_T_ that represent the inputs of the C and T states. At very low input levels, the internal noise of the system is much stronger than the input strengths and thus makes the system oscillate much faster between the two states. This effect is in line with our hypothesis that lower grating contrasts would increase the number of switches and percept durations, which led us to perform Experiment 2 with the idea that AMB should exhibit an increase in the number of switches and also show a decrease in the durations of the percepts. However, the results differed from our expectation, with NTE showing the predicted effect, but AMB showing no changes with lower grating contrasts.

## Discussion

We investigated putative differences in the visual motion system between anisometropic amblyopes and neurotypical observers through the use of bistable plaid motion perception. First, our group of amblyopes globally exhibited normal bistable perception in any viewing condition (binocular, monocular with amblyopic or fellow eye) when compared to the control group. Second, we hypothesized that lower contrast of the plaid stimulus should emphasize the internal noise differences between the two groups and thus lead to a stronger increase in percept switches and decrease in percept durations. The results confirmed this hypothesis only in the control group, while the amblyopic group exhibited no changes. These latter results are at odds with the idea of stronger noise in the amblyopic motion system, and plausible explanations of these discrepancies are discussed below.

Bistable perception of plaid square gratings was found to be normal in anisometropic amblyopes when compared to that in the neurotypical controls. These results are in agreement with previous reports of normal perception of bistable sine-grating plaids in such group of subjects (Thompson et al., 2008, 2012; Hamm et al., 2014), even when first-order contrast deficits are taken into account (Tang et al., 2012). In our study, these earlier reports are confirmed through analysis of perceptual bistability applied on square gratings.

While bistability of the percepts was similarly seen and stochastic across eye-viewing conditions and groups of subjects, our methods and results unveiled a new and unexpected effect of contrast on plaid motion perception in amblyopes. Based on reports of possibly stronger internal noise in the amblyopic visual motion system (Simmers et al., 2003; Mansouri and Hess, 2006; Hamm et al., 2014) and theoretical insights into perceptual bistability and neural noise (Brascamp et al., 2006; Moreno-Bote et al., 2007; Shpiro et al., 2009; Huguet et al., 2014), lower contrasts of the stimulus were argued to decrease the duration of each percept in amblyopes when compared to that in the control group. This effect was found, but it was reversed between groups, with the control group showing decreased percept stability (decrease in percept durations), while the amblyopes did not exhibit such an effect.

This result is interesting in at least two aspects. First, contrast sensitivity, the reciprocal of contrast threshold that is used to describe subjects' ability to visually detect a target, is known to be strongly affected in amblyopic eyes (Woodruff, 1991). Earlier research has shown that contrast sensitivity is highly decreased in the amblyopic eye, especially at high spatial frequencies, but the sensitivity of the fellow eye is also affected when compared with the eyes in normal subjects (Bradley and Freeman, 1981). Interestingly, amblyopes do not exhibit clear deficits in contrast perception at suprathreshold stimulus contrasts, indicating that there is no clear contrast coding abnormality for the suprathreshold contrast range in amblyopes (Hess and Bradley, 1980; Loshin and Levi, 1983). On the contrary, suprathreshold static grating perception is affected but in a very different manner. Amblyopes staring at images of classic square gratings perceive perceptual distortions of the stimulus that could be of static or dynamic nature (Hess et al., 1978; Sireteanu et al., 2008; Thiel and Iftime, 2016). Thus, the two facts that (1) our group of amblyopes perceived the 120-s moving plaids normally, with classic perceptual bistability and no reports of differences in perception between the weak and fellow eyes, and (2) amblyopes did not show an effect of contrast on the global bistability of the percept hint to a motion coding system in their visual pathway that uses dynamic visual input in a different way from neurotypical subjects. The results of neurotypical subjects experimentally confirmed the inversed “Levelt IV rule” at low contrasts (Brascamp et al., 2015), but the overall pattern of results led us to consider in further detail the models of plaid motion perception and a plausible explanation of the effects observed in amblyopes.

In analyzing and applying a model (Huguet et al., 2014), we found that input intensity indeed affected percept switches and durations as hypothesized. These effects also suggested that, for amblyopes, contrast of the stimulus is decoupled from or very weakly related to the “input” variable of the model. This suggests that there may be different motion coding system in the amblyopic visual system from that in the neurotypical one, with the perceptual switches observed in the former visual motion system related to different mechanisms.

From a neurophysiological perspective, motion coding and decoding of plaid stimuli might not be performed at a single stage, but instead, multiple areas may be involved (Thompson et al., 2012; Villeneuve et al., 2012). Thus, the segregation of motion (transparency) or the assimilation of motion (coherency) may be coded in a distributed manner across the early cortices. The differences between our amblyopic and control groups in contrast effects might stem from the fact that, in the amblyopic system, motion coherency and transparency coding could be more widely distributed than in neurotypical subjects, as suggested by a recent study (Thompson et al., 2012). From a different and more detailed perspective, the major motion area MT is known to contain cells that can selectively respond to the pattern or components of moving plaid gratings (Rust et al., 2006) and, furthermore, has some depth coding structure (Born and Bradley, 2005) that should help to create depth ordering of different motion surfaces. Although MT cells in the macaque monkey seem to have dominance over fellow eye inputs, the distribution of cells sensitive to pattern and the components of plaid gratings were found equal (El-Shamayleh et al., 2010), thus showing global similar plaid motion coding. Therefore, we might assume that the equivalent percepts of coherence and transparency are decoded through a simple rule: to decode only one neuronal population—component or pattern cells. Because MT cells receive major input from V1 cells, the contrast dependence of all MT cells should be similar. The observation in control subjects of stronger perceptual changes at lower contrast supports the idea that pattern and component cells should be similarly activated by contrast strength. On the other hand, the lack of contrast effects in amblyopes seems to indicate that pattern and component cells have different input relations to the contrast of the stimulus. This difference provides an interesting possibility and its exact nature is far from the scope of the current study.

Importantly, the model used here is more qualitative in nature, helping to grasp essential structural differences and changes in the multistable perception of plaid motion stimuli but not providing a realistic implementation of motion coding. Recent studies reported that, closely related to our work, tristable motion perception could be explained by a more detailed motion-tuned neuronal population (Meso et al., 2016; Medathati et al., 2017) that more closely resembles MT physiology. Further investigations and theoretical modeling also incorporating depth coding should help to unravel the plausible changes in the amblyopic motion system.

A systematic and interesting difference we found was the global direction effect. Both amblyopes and normal subjects had more percept switches when global motion direction was upward, i.e., vertical, than when it was horizontal. We did not find systematic effects between the two groups across the first two experiments. Differences between cardinal axes have already been reported in previous studies of visual motion perception in ambiguous conditions (Castet et al., 1999; Hupé and Rubin, 2004). The exact nature of the asymmetry in bistability between vertical and horizontal global motions may lie in the eye movement differences between these two cardinal directions. The global effect present across all observers might stem from clear differences in eye movement dynamics of horizontal and vertical eye movement (fixational, reflexive, or voluntary pursuit eye movements) (Baloh et al., 1988; Sparks, 2002). This explanation partly supports a separate control of vertical and horizontal pursuit, which may contribute to the direction difference that is systematically reported. Furthermore, eye movement may influence the percept through retinal motion. Van Dam et al. demonstrated that the retinal image shift, caused by saccade, can change the bistable percept (van Dam and van Ee, 2005, 2006). For clarification of the exact mechanism of such a direction effect and determination of whether amblyopes with clear changes or deficits in eye movements exhibit an effect on perception of plaid motion, further studies are still needed with proper measures and controls for eye movements in neurotypical and amblyopic groups.

In summary, by using bistable plaid motion as a probe of the visual motion system, we found a systematic and clear effect of stimulus contrast on perceptual bistability in neurotypical subjects that was not present in anisometropic amblyopes. The former effect is explained by classic models of multistability and thus hints toward a generally different motion coding and decoding system in the amblyopes.

## Ethics statement

This study was carried out in accordance with the recommendations of Ethical review of biomedical research involving human beings, Committee on biomedical ethics of university of science and technology of China with written informed consent from all subjects. All subjects gave written informed consent in accordance with the Declaration of Helsinki. The protocol was approved by the Committee on biomedical ethics of university of science and technology of China.

## Author contributions

JL carried out the experiments, and wrote the manuscript with the support of TT and YZ. Both JL and TT contributed to the design and implementation of research, to the analysis of results. YZ supervised the project. All the authors reviewed the manuscript and conceptualized this study.

### Conflict of interest statement

The authors declare that the research was conducted in the absence of any commercial or financial relationships that could be construed as a potential conflict of interest.
